# Cannabidiol in sports: insights on how CBD could improve performance and recovery

**DOI:** 10.3389/fphar.2023.1210202

**Published:** 2023-09-22

**Authors:** Daniel Rojas-Valverde, Andrea Fallas-Campos

**Affiliations:** ^1^ Sport Injury Clinic (Rehab Readapt), Escuela Ciencias del Movimiento Humano y Calidad de Vida (CIEMHCAVI), Universidad Nacional, Heredia, Costa Rica; ^2^ Núcleo de Estudios para el Alto Rendimiento y la Salud (CIDISAD-NARS), Escuela Ciencias del Movimiento Humano y Calidad de Vida (CIEMHCAVI), Universidad Nacional, Heredia, Costa Rica; ^3^ Núcleo de Estudios para el Alto Rendimiento y la Salud (ACUAUNA-NARS), Escuela Ciencias del Movimiento Humano y Calidad de Vida (CIEMHCAVI), Universidad Nacional, Heredia, Costa Rica

**Keywords:** cannabis, THC, rest, stress, training, anti-inflammatory, ergogenic aid

## What is cannabidiol (CBD)?

Cannabidiol is popularly known as CBD, a substance that is part of the cannabinoids, chemical components extracted from the cannabis or hemp plant. Of all the chemical substances extracted from cannabis, some are legal, and others are not. CBD’s consumption, sale, and distribution are permitted and legal in some countries worldwide, such as the United States, Spain, Germany, China, Uruguay, Costa Rica, and Morocco.

The World Anti-Doping Agency, the institution controlling prohibited substances in sports worldwide, has accepted CBD among professional athletes (Nichols and Kaplan, 2019). Normally, CBD can be consumed in multiple products, in drops of oil, processed foods, drinks and other products (Lim et al., 2020) that athletes can find in a supermarket or specialised sports store. For this reason and its apparent benefits, the consumption of CBD has increased significantly among athletes (Docter et al., 2020). This has fueled a race to study its properties, benefits and risks for the health and performance of athletes.

Coaches, athletes, doctors, therapists, and scientists are constantly concerned with finding ways to improve the performance of athletes by making athletes faster, more resistant, more agile, rest and recover better from efforts and feel better. Athletes try a series of substances, technologies, and training methodologies to win (Bampouras et al., 2012). In the case of CBD, the studies that have been carried out so far are insufficient to adjudicate ergogenic, ergolytic, and there is a lack of experimentation in humans, especially in its effects on athletes and physically active people (Kennedy, 2017; Maurer et al., 2020; McCartney et al., 2020; Burr et al., 2021; Rojas-Valverde, 2021). Despite this lack of knowledge on the effects on athlete’s performance and health, based on its impact on other populations and health problems, some potential benefits should be more in-depth analysed.

**FIGURE 1:**
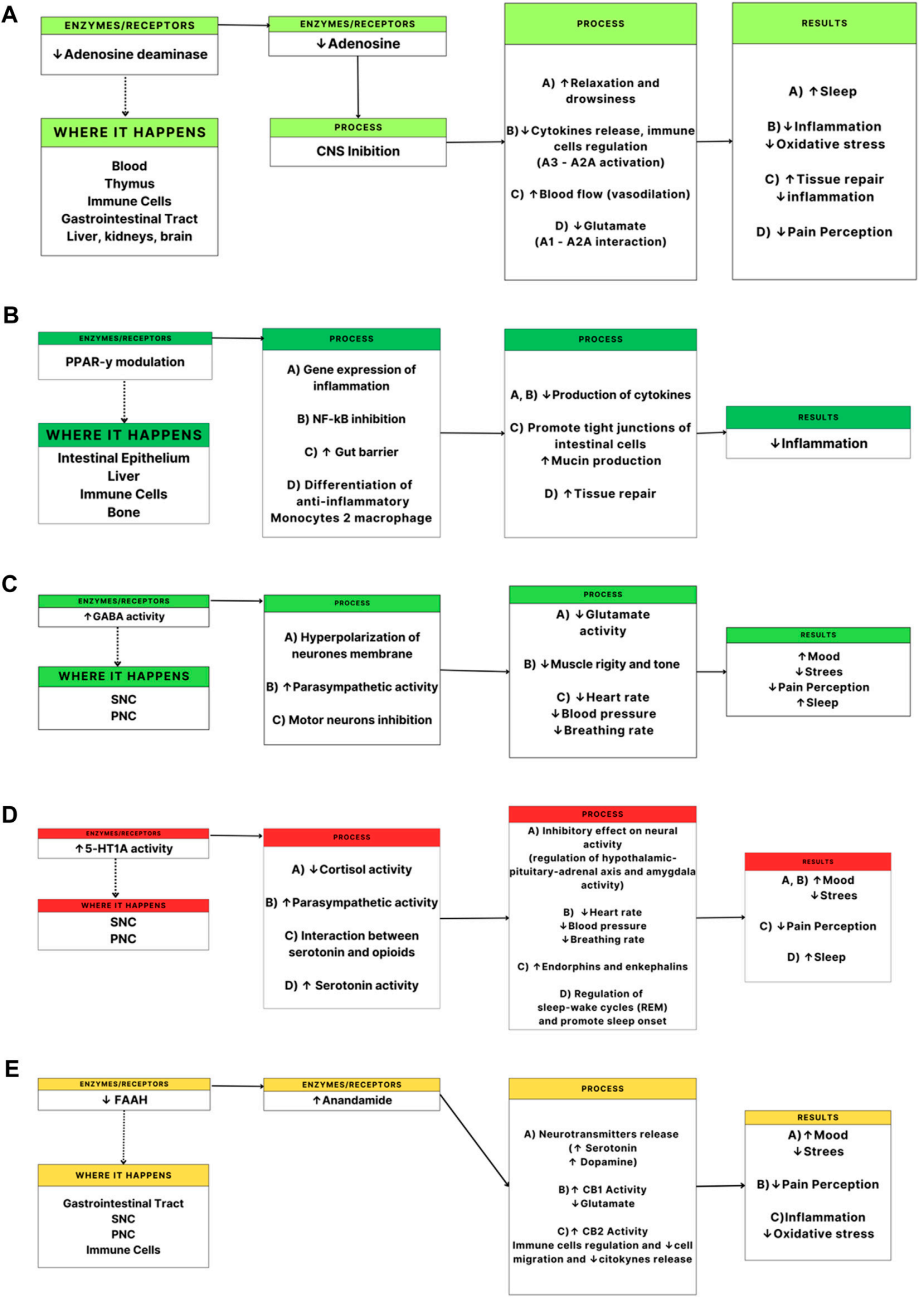
Potential benefits of cannabidiol (CBD) intake on sports performance and recovery: physiological pathways and enzymes and receptors interaction. **(A)** adenosine deaminase, **(B)**. PPAR-*y*: gamma peroxisome proliferator-activated receptors, **(C)**. GABA: gamma-aminobutyric acid, **(D)**. 5-HT1A: 5-hydroxytryptamine receptor 1A, **(E)**. FAAH: fatty acid amide hydrolase. CNS: central nervous system, PNS: peripheral nervous system.

Based on what is currently known, CBD has potential benefits and properties that could help the athlete feel better when facing competition (Kennedy, 2017; McCartney et al., 2020; Rojas-Valverde, 2021). Among these benefits, the consumption of CBD could make athletes rest better (e.g., improve sleep latency, sleep continuity, subjective sleep quality and reduce nightmares and insomnia) (Russo et al., 2007; Choi et al., 2020; Mondino et al., 2021; Ranum et al., 2023), reduce their stress and feel better in the face of competition and training (anxiolytic and antidepressant) (Narayan et al., 2022), can deflate their muscles after the damage caused by physical exertion (anti-inflammatory) (Kennedy, 2017; Gamelin et al., 2020; Villanueva et al., 2022; Stone et al., 2023), and reduce pain caused by high physical demands (pain and soreness reliever) (see Figure 1) (Kennedy, 2017; Gamelin et al., 2020; Henson et al., 2022).

## What causes CBD in the body of athletes?

CBD is a natural substance that causes changes and alterations at the physiological and cognitive (mental and emotional) levels (Stout and Cimino, 2014). These changes appear because CBD influences the function of an endocannabinoid system, which is responsible for maintaining homeostasis (Nichols and Kaplan, 2019). This system participates in processes related to neurogenesis, brain plasticity, control mode, dopamine release, and fatty acid hydrolase release. These functions, therefore, regulate how we feel emotionally, how the brain learns and multiplies its nerve connection networks, controls inflammation (anti-inflammatory) and how we perceive pain (analgesic) (Rojas-Valverde, 2021). CBD intake increases oxygen consumption and pleasure ratings during endurance running (Sahinovic et al., 2022). Also, preclinical studies have shown how CBD could protect myocardial injury during intense exercise, demonstrating anti-inflammatory, anti-apoptosis and antioxidative stress effects (Zhang et al., 2022).

The cannabis system enables numerous effects during physical exertion, including sensations of joy, calm, and euphoria (Carek et al., 2011). Endocannabinoids, such as anandamide and 2-arachidonoylglycerol (2AG), behave as cannabinoids by activating cannabinoid receptors called type-1 (CB1) and type-2 (CB2) receptors. These molecules, comparable to N-acyl ethanolamine’s (De Petrocellis and Di Marzo, 2009), generate benefits similar to exercises, such as hunger control, inflammation reduction, anxiety relief, and prevention of excessive cell proliferation. CBD inhibits the degradation and absorption of endocannabinoids such as anandamide, increasing endocannabinoids' binding to their receptors. CB1 receptors are located in the central nervous system, whereas CB2 receptors are found in the peripheral nervous system.

Ccannabinoids and endocannabinoids are required for the release of brain-derived neurotrophic factor, which aids in processes such as neurogenesis and neural plasticity. They also play a role in releasing glucocorticoids, which help regulate mood by alleviating symptoms of melancholy and anxiety. Cannabis also stimulates dopamine release, resulting in a sensation of pleasure. Furthermore, they are linked to fatty acid amide hydrolase release, which results in analgesic effects. Notably, these reactions are consistent with the beneficial effects of exercise (Tantimonaco et al., 2014). Stimuli that activate TRPV1 ion channels (Vanilloid receptors) cause these actions, which result in antinociceptive effects (Gochman et al., 2023). Stimuli targeting CB1 and CB2 receptors elicit relaxation through neurodepression and cytokine release inhibition, respectively (Jean-Gilles et al., 2015). Furthermore, the stimulation of 5HT1A receptors promotes serotonin absorption in postsynaptic neurons, which helps to regulate mood states (Resstel et al., 2009). Figure 1 is an in-depth representation of the potential physiological pathways and the interactions between enzymes and receptors with CBD in the human body.

New evidence has suggested that in humans, CBD intake could improve satellite cell differentiation in muscles, improving muscle recovery (e.g., muscle damage attenuation) and performance (e.g., strength) (Schouten et al., 2022). Also, recent findings demonstrate modest yet meaningful effects on muscle damage and recovery (reduction in creatine kinase and myoglobin) within a 72-h after 60 mg of CBD supplementation (Isenmann et al., 2021). The evidence is contradictory in this sense, and the debate is more open than ever (Cochrane-Snyman et al., 2021; Crossland et al., 2022; Stone et al., 2023), which is why more quantity, quality and variety of specific studies on sport and exercise are necessary. This recent data gives promissory insights on using CBD as a performance enhancer and recovery aid, even though serious doubts about its use (e.g., dose administration) and safety must be carefully addressed.

## CBD to improve sleep quality

Athletes frequently overreact because of high training loads and inadequate recovery between efforts. These conditions can cause sleep disturbances or moments in which the athlete cannot rest comfortably, impacting sleep quality or recovery. CBD appears to regulate the cycle in which the body stays awake or asleep, which is essential for an athlete’s recovery (Burstein, 2015; Hill et al., 2017). One of the advantages of CBD consumption is its potential to enhance sleep in athletes. This includes improvements in sleep initiation, uninterrupted sleep, subjective sleep quality, as well as a reduction in nightmares and insomnia symptoms (Russo et al., 2007; Choi et al., 2020; Mondino et al., 2021; Ranum et al., 2023). In addition, some substances promote sleep controlled by the endocannabinoid system, which we can activate by consuming CBD (McCartney et al., 2020; Rojas-Valverde, 2021).

Sleep management requires a precise balance of neurotransmitters, and CBD’s actions on the endocannabinoid system contribute to this balance. CBD interacts with adenosine receptors, which is significant since adenosine is a neurotransmitter that promotes sleep and relaxation. CBD promotes tranquillity and preparedness for sleep by boosting adenosine signalling. Furthermore, CBD’s effect on GABAergic neurotransmission adds to its sleep-enhancing properties (Kesner and Lovinger, 2020; Kaul et al., 2021). GABA is an inhibitory neurotransmitter that promotes relaxation and drowsiness by lowering neuronal excitability. CBD’s effect on GABA receptors can promote deeper, more comfortable sleep. Furthermore, CBD’s ability to relieve anxiety and stress, which are significant causes of sleep disruption, indirectly supports greater sleep quality (Blessing et al., 2015; Moltke and Hindocha, 2021; Ortiz Rios et al., 2022). CBD provides a biological foundation for its action via modifying endocannabinoid system signalling, increasing adenosine effects, and regulating GABAergic neurotransmission (Zou & Kumar, 2018; Yarar, 2020; Martinez Naya et al., 2023).

## CBD to reduce stress and regulate mood

Usually, due to athlete’s significant effort during their sports practice, they suffer from fatigue, which can lead them to situations where they do not feel very well emotionally. The ability of CBD to regulate the athlete’s mood is being studied (Kasper et al., 2020).

CBD can boost anandamide signalling, an endocannabinoid related to emotions of wellbeing, by preventing its absorption and breakdown, resulting in higher levels in the brain (Leweke et al., 2012; Henson et al., 2022). CBD has also been demonstrated to interact with serotonin receptors, including the 5-HT1A receptor, which regulates mood. Research findings indicate that CBD has been found to decrease anxiety levels by activating the 5-HT1A receptors and restoring impaired neurotransmission of the 5-HT1A (serotonin) system (De Gregorio et al., 2019). CBD can help serotonin transmission by attaching to these receptors. Serotonin is a neurotransmitter that is directly tied to mood and emotions. Furthermore, CBD has been shown to influence the hypothalamic-pituitary-adrenal axis, a critical mechanism in the body’s stress response. CBD reduces stress response by inhibiting the production of stress hormones such as cortisol. Overall, CBD’s capacity to modify endocannabinoid system function, increase anandamide signalling, interact with serotonin receptors, and influence stress hormone release all contribute to its potential for pain relief (Viudez-Martínez et al., 2018; Yarar, 2020; Lookfong et al., 2023).

CBD effects on anxiety seem to depend on dosage; 300 mg is more effective than 150 or 600 mg for reducing anxiety-related symptoms (Linares et al., 2019). There is no evidence of reduced anxiety or mood regulation in sports. Still, it seems that CBD could have certain properties that can be anxiolytic and anti-depressive (Murillo-Rodríguez et al., 2020) that some athletes suffer due to the pressure they always have to be better and win, as well as the frustration they may suffer from not achieving certain goals (McCartney et al., 2020; Rojas-Valverde, 2021).

## CBD to reduce inflammation and oxidative stress

Inflammation and oxidative stress are two processes that intervene in people’s general health (McPartland et al., 2015). These two processes are normally triggered after exercise in athletes, and as we can control them, the athlete will feel more recovered and be more prepared to exert effort again. Inflammation is caused because, during exercise, the muscles suffer tension that causes damage, and by becoming inflamed, the body initiates the processes to repair that damage (McCartney et al., 2020; Rojas-Valverde, 2021).

Inflammation is necessary to recover from significant efforts. Still, excess inflammation could cause problems in our digestive and musculoskeletal systems and other systems due to the damage to tissues and organs that this causes (McCartney et al., 2020); that is why controlling it is optimal. CBD in athletes could regulate inflammatory processes by reducing substances that usually cause unwanted increases in inflammation, such as cytokines and cortisol (Zuardi et al., 1993). In addition to muscle and digestive inflammation, CBD reduces oxidative stress and neuroinflammation (Atalay et al., 2019; Sahinovic et al., 2022). In this regard, 300 mg of CBD has been shown to induce glucocorticoid regulation, such as cortisol in humans, a key regulator of the inflammatory response to injury (Zuardi et al., 1993).

Based on recent evidence, 10 mg/kg of CBD could attenuate inflammation (e.g., IL-6, IL-1 and tumour necrosis factor *α*) after fatiguing eccentric exercise by activating cannabinoid receptor two (Stone et al., 2023). This is based on CBD’s interactions with inflammation-controlling receptors (CB1 cannabinoid, CB2 cannabinoid, adenosine A2A), its cytokine level-reducing actions, and its moderation of immune cell activity, thus mitigating collateral tissue inflammation (Booz, 2011; Burstein, 2015; A. J; Hill et al., 2012). Moreover, CBD’s potential to enhance the release of arachidonic acid could improve healing by regulating growth signals mediated by pro-resolving substances (e.g., lipoxin A4 and 15d-PGJ2) (Burstein, 2015).

## CBD to reduce the pain

CBD appears to have analgesic properties and bone that can decrease pain (Marques Azzini et al., 2023). Due to exercise, athletes usually feel pain from the effort and the damage caused to their bodies when they reach the limit. Running, pedalling, jumping, changing directions, hitting, and kicking generate muscle breakdown that causes inflammation, which can become painful.

For example, Sativex, THC, and CBD have been licensed to treat central and peripheral neuropathic pain. This pain condition is linked to activated microglia and a subsequent cascade of proinflammatory cytokines, including IL-6, IL-1, and TNF (Booz, 2011). In addition to its neuroprotective properties, this effect was discovered in a recent systematic analysis of the result of CBD consumption in connection to its prospective usage as a performance-enhancing agent (McCartney et al., 2020). It is currently unknown how CBD interacts with the pain cascade and pathways (Anthony et al., 2020). Still, it is suggested that serotonin and opioid interactions could have a great role in endorphins and enkephalins release and reduction of glutamate release via the interaction of adenosine 1 and A2A, leading to pain reduction (Navarrete et al., 2021; Peng et al., 2022). CBD has demonstrated its ability to cure and control pain in illnesses and pain disorders, and based on this information, CBD appears to have a possible effect on reducing swelling and avoiding soreness after hard activity (Sahinovic et al., 2022), but further research is needed to make a definitive declaration.

CBD, in a specific manner, interferes with neuronal communication, preventing the transmission of information related to pain (e.g., inhibition of neurotransmitter activity). As a result, the pain sensation is not perceived as it typically would be (McCartney et al., 2020; Rojas-Valverde, 2021). There is evidence of using CBD for chronic and acute pain management (Alaia et al., 2022; Marques Azzini et al., 2023). CBD can promote analgesia by activating transient receptor potential cation channel subfamily V (TRPV1) and serotonin receptors (Naik and Trojian, 2021). The latest scientific data found a pain-reliever effect of topical application (2*10 mg/day) of CBD in elite athletes with only minor side effects (e.g., dry skin) (Hall et al., 2023).

## What care should we have, and what remains to be demonstrated scientifically?

We must be careful to consume CBD products that official health institutions approve. Because CBD is illegal in certain countries, it is normal to find products with other substances that can cause unwanted side effects or could represent a legal issue for athletes. Concerns around athlete doping are raised because certain CBD products include THC and other cannabinoids (Hazekamp, 2018; Evans, 2020; Johnson et al., 2022). When utilising CBD products, athletes should take caution and make sure they are using reliable, independently tested goods that verify there is no THC or other illegal cannabinoids present.

In addition, it is important to consider that CBD is unlike any other food, so the amount we consume must be regulated. Scientists are still unsure how much dose is needed to cause certain reactions in the body (McCartney et al., 2020; Rojas-Valverde, 2021). Also, recent evidence in humans still shows highly variable dosing and methodological concerns that should be addressed when consuming CBD products (Schouten et al., 2022). In exercise and sport-related evidence, the dose could be a key in finding performance or recovery benefits. For example, 2 and 5 mg/kg seem ineffective for these purposes, but 10 mg/kg is (Crossland et al., 2022; Stone et al., 2023), even higher doses of CBD (25 mg/kg) seem secure for consumption in humans and its effects could be studied in future studies (Grotenhermen et al., 2017). Also, the drug-drug interaction of CBD with other drugs should be explored when used for athletic purposes (Lopera et al., 2022). When discussing and advocating the use of CBD, professionals working with the sports community must consider any potential legal, medical, and ethical concerns.

## Future research recommendations

With the growing interest in the use of CBD in athlete recovery, more research is warranted to understand its physiological mechanism of action, potential benefits, and intended safety and efficacy profile when consuming CBD before, during, and after training or competition. Future sports science and medicine research should focus on understanding the role of CBD in physiological mechanisms such as the inflammatory cascade, neuroprotection, analgesic and anxiolytic pathways, muscular enhancement, and neuromechanical function.

New randomised controlled trials with a placebo should consider different fatigue and damage etiologies, individualities, disciplines, needs and special characteristics. Other potential research areas include optimal dosing based on physical and physiological load, efficacy concerning administration timing, chronic and acute effects, cumulative responses with different recovery strategies, differences in tolerance and effectiveness by sex, professional level, fitness level, and other individual conditions and situational factors. Furthermore, more information is needed to understand CBD’s inflammatory signalling as an essential factor in the recovery process. The effectiveness of CBD compared to conventional medications should be evaluated.

## Conclusion

CBD appears to have anti-inflammatory, neuroprotective, analgesic, anxiolytic, and potentially recovery-mediating properties in athletes, but more scientific evidence is needed to confirm these effects. Confirmatory analyses using randomised controlled trials with placebo are necessary to test the acute and chronic effects of different dosage prescriptions. These studies must consider fundamental sport-specific particularities, such as the diverse biological and situational conditions that contribute to fatigue, the characteristics of each discipline during training and competition, the individual peculiarities of athletes, their tolerance and response to CBD intake, and the combined effect of CBD administration with other physical and nutritional aids.

Given the relatively common use of cannabis and CBD among athletes, there is a clear need to improve the scientific understanding of the effects of CBD use on athlete recovery and performance. Further scientific progress is necessary, primarily through the execution of experimental trials, to better understand critical positive and negative outcomes for the ultimate benefit of athlete recovery and performance. Furthermore, resulting evidence could provide new clinical guidance for prescribing CBD during the athlete recovery process and other potential applications. The potential therapeutic benefits of CBD administration have been minimised for years, but the actual scenario could increase knowledge about this natural compound and its effects. Additionally, from an administrative point of view, adopting a clearer and more global policy for the use of cannabis in sports should be considered.
